# Nuclear translocation of HIF-1α induced by influenza A (H1N1) infection is critical to the production of proinflammatory cytokines

**DOI:** 10.1038/emi.2017.21

**Published:** 2017-05-24

**Authors:** Xinkun Guo, Zhaoqin Zhu, Wanju Zhang, Xiaoxiao Meng, Yong Zhu, Peng Han, Xiaohui Zhou, Yunwen Hu, Ruilan Wang

**Affiliations:** 1 Department of Critical Care Medicine, Shanghai General Hospital, Shanghai Jiaotong University School of Medicine, Shanghai 200080, China; 2 Department of Pathogen Diagnosis and Biosafety, Shanghai Public Health Clinical Center, Key Laboratory of Medical Molecular Virology, Ministry of Education and Health, Shanghai Medical College, Fudan University, Shanghai 201508, China

**Keywords:** H1N1, HIF-1α, hypoxia, inflammation, nuclear translocation

## Abstract

Infection with the influenza A (H1N1) virus is a major challenge for public health because it can cause severe morbidity and even mortality in humans. The over-secretion of inflammatory cytokines (cytokine storm) is considered to be a key contributor to the severe pneumonia caused by H1N1 infection. It has been reported that hypoxia-inducible factor 1-alpha (HIF-1α) is associated with the production of proinflammatory molecules, but whether HIF-1α participates in the acute inflammatory responses against H1N1 infection is still unclear. To investigate the role of HIF-1α in H1N1 infection, the expression and nuclear translocation of HIF-1α in A549 and THP-1 cell lines infected with H1N1 virus were observed. The results showed that without altering the intracellular mRNA or protein expression of HIF-1α, H1N1 infection only induced nuclear translocation of HIF-1α under normal oxygen concentrations. The use of 2-methoxyestradiol (2ME2), a HIF-1α inhibitor that blocks HIF-1α nuclear accumulation, in H1N1-infected cells decreased the mRNA and protein expression of tumor necrosis factor-alpha (TNF-α) and interleukin (IL)-6 and increased the levels of IL-10. In contrast, H1N1-infected cells under hypoxic conditions had increased HIF-1α nuclear accumulation, increased expression of TNF-α and IL-6 and decreased levels of IL-10. In conclusion, our data implied that *in vitro* H1N1 infection induced nuclear translocation of HIF-1α without altering the expression of HIF-1α, which may promote the secretion of proinflammatory cytokines during H1N1 infection.

## INTRODUCTION

Since it first emerged in Mexico in the early 2009,^1^ H1N1 viral infections have been recorded in most areas of the world^2, 3, 4^ and are an important cause of severe acute pneumonia, which often results in acute respiratory distress syndrome (ARDS), a syndrome with a high risk of mortality.^5, 6, 7^ Therefore, H1N1 infection represents a severe challenge and a significant threat to public health.

Many factors may complicate the severe respiratory syndrome caused by influenza virus infection, of which, the cytokine storm is a key contributor in modulating the immune response.^8, 9^ High levels of proinflammatory cytokines were characterized in the sera of patients infected with the H1N1, H5N1 and H7N9 influenza viruses.^9, 10, 11^ Proinflammatory cytokines, such as tumor necrosis factor-alpha (TNF-α) and interleukin (IL)-6, promote inflammation and directly damage the alveolar–capillary membrane, leading to edema and surfactant inactivation.^12, 13^ Diffused inflammation and damage of alveolar and capillary lung structures can cause progressive hypoxemia, eventually leading to ARDS.^14^

Hypoxia-inducible factor (HIF) is a heterodimeric transcription factor that is composed of a HIF-1α (or its analogs, HIF-2α and HIF-3α) and a HIF-1β subunit.^15^ Control of HIF-1 is mainly achieved by regulating the expression of HIF-1α, an oxygen sensitive subunit, which can be upregulated rapidly in response to hypoxia.^16^ HIF-1α is a key mediator in cell metabolism, angiogenesis and inflammation.^17, 18, 19^ Recent studies have focused on the role of HIF-1α in the regulation of inflammation. HIF-1α is involved in the transcriptional regulation of cytokines, such as IL-6 and TNF-α, during inflammation.^20, 21, 22^ In addition, data suggest that HIF-1α expression can be induced by virus infection.^23, 24, 25, 26^ However, the role of HIF-1α in H1N1 infection is still unknown.

We investigated the expression and function of HIF-1α in response to H1N1 infection. We found that at a normal concentration of oxygen, instead of altering the mRNA and total protein levels of HIF-1α *in vitro,* H1N1 infection induced nuclear translocation of HIF-1α, which may play a significant role in inflammation.

## MATERIALS AND METHODS

### Reagents and antibodies

2-Methoxyestradiol (2ME2) was purchased from Selleck (Houston, TX, USA). RPMI 1640, Ham's F-12K and fetal bovine serum were purchased from Gibco/BRL Life Technologies (Grand Island, NY, USA). Anti-HIF-1α primary antibodies were purchased from BioWorld (St Louis Park, MN, USA). Lamin B1 antibodies were obtained from Thermo Fisher Scientific (Waltham, MA, USA). Anti-β-actin primary antibodies were obtained from Cell Signaling Technology (Boston, MA, USA). Their respective horseradish peroxidase (HRP)-conjugated secondary antibodies were purchased from Beyotime (Shanghai, China). PVDF membranes were purchased from Bio-Rad (Richmond, CA, USA). The enhanced chemiluminescence (ECL) agent was obtained from Thermo Fisher Scientific (Waltham, MA, USA). An Immunol Fluorescence Staining Kit with Alexa Fluor 555-Labeled Donkey Anti-Rabbit IgG and 2-(4-amidinophenyl)-6-indolecarbamidine (DAPI) were obtained from Beyotime (Shanghai, China). TRIzol was purchased from Invitrogen (Grand Island, NY, USA), and One Step PrimeScript RT-PCR Kit was obtained from TaKaRa (Dalian, Liaoning, China).

### Cell cultures and viruses

Human lung adenocarcinoma epithelial cells (A549) and human acute monocytic leukemia cells (THP-1) were purchased from the American Type Culture Collection (Rockville, MD, USA). A549 cells were cultured in Ham’s F-12K with 10% fetal bovine serum (Gibco) and 1% antibiotics (100 U/mL penicillin, 0.1 mg/mL streptomycin). THP-1 cells were cultured in RPMI 1640 with 10% fetal bovine serum and 1% antibiotics. All cells were cultured in an incubator at 37 °C and 5% CO_2_. The hypoxia condition was generated using 1% O_2_, 5% CO_2_, and 94% N_2_ in a tri-gas incubator. The influenza A virus strain, A/PR/8 (H1N1), was obtained from ATCC. A549 and THP-1 cells were inoculated with PBS (control) or infected with H1N1 at a multiplicity of infection (MOI) of 0, 0.1, 0.5, 1, 2, and 4 and 0, 1, 2, 5, 10 and 15, respectively, and the virus was diluted in culture solution. After 4 h, the inoculum was removed and the cells were washed twice with PBS before new culture solution was added. To block the translocation of HIF-1α, A549 and THP-1 cells were pretreated with 2ME2 for 30 min. The concentrations of 2ME2 were determined using a cell viability assay (Supplementary Figure S1).

### Western blot

Total cellular protein from cultured cells was extracted with RIPA buffer (Beyotime Biotechnology, Shanghai, China), and the total protein concentration was determined using a BCA protein assay kit. NE-PER nuclear and cytoplasmic extraction reagents (Thermo Fisher, Rockford, USA) were used as per the manufacturer’s instructions to extract nuclear and cytoplasmic proteins from the cells. Typically, 15–55 mg of total protein was separated using an 8% SDS-PAGE gel and transferred onto a PVDF membrane. The membranes were then blocked with 5% non-fat milk and incubated overnight at 4 °C with primary antibodies specific to HIF-1α (1:500, BioWorld Technology, USA), Lamin B1 (1:1000, Thermo Fisher, Rockford, USA), and β-actin (1:1000, Cell Signaling Technology, USA). After washing three times for 10 min each with 15 mL of TBST, the membranes were incubated with HRP-conjugated secondary antibodies (1:2000) for 1.5 h at room temperature. The membranes were then washed again, three times for 10 min each with 15 mL of TBST. Finally, the protein bands were visualized using ECL reagent (Millipore, USA).

### ELISA

Supernatants from cultures of A549 and THP-1 cells infected with H1N1 for 24 h at MOIs of 1 and 5, respectively, were collected and the concentrations of IL-6, IL-10 and TNF-α in the supernatants were measured according to the ELISA kit manufacturer’s instructions (R&D Systems, USA).

### Immunofluorescence analysis

THP-1 cells were treated with H1N1 at an MOI of 5 for 24 h. After 24 h, cells were collected by centrifugation, and the cells were fixed for 10 min using 4% paraformaldehyde at room temperature. Then, the cells were smeared onto polylysine-coated slides (Hehy, Jiangsu, China). After the paraformaldehyde was volatilized, the slides were washed five times with PBS for 3 min each time and permeabilized with 0.5% Triton X-100 in PBS for 10 min at room temperature. The slides were blocked for 60 min at room temperature in 5% bovine serum albumin, incubated overnight at 4 °C with HIF-1α antibody (1:50 dilution), washed five times with PBS for 3 min each time and incubated for 60 min at room temperature with Alexa Fluor 555-Labeled Donkey Anti-Rabbit IgG (1:250 dilution, Beyotime Biotechnology, Shanghai, China). Nuclei were stained with DAPI for 3 min and then the cells were washed and observed under a laser confocal scanning microscope (Leica, Wetzlar, Germany). A549 cells were grown in confocal dishes and treated with H1N1. After 24 h, the cells were washed twice with PBS and fixed for 10 min using 4% paraformaldehyde at room temperature. The steps outlined above were then followed.

### RNA isolation and real-time PCR

Total RNA was extracted from cells using TRIzol (Invitrogen, Carlsbad, CA, USA) according to the manufacturer’s protocol. The concentration of total RNA was quantified using an ultraviolet spectrophotometer (ND-1000 spectrophotometer; NanoDrop Technologies, Wilmington, DE). Quantitative real-time PCR (qRT-PCR) was performed using a One Step PrimeScript RT-PCR Kit from TaKaRa (Dalian, Liaoning, China) on the Vii7 system (ABI, Carlsbad, CA, USA). The primer sequences are listed in Supplementary Table S1. Quantification was finished using a Real-Time One Step RT-PCR protocol as described in the manual. The protocol consisted of 1 cycle at 42 °C for 5 min and 95 °C for 10 s, 40 cycles at 95 °C for 5 s, and 1 cycle conducted at 60 °C for 31 s, at which point fluorescence data were collected. The relative change in mRNA was normalized to β-actin and calculated using the 2^−ΔΔCT^ method. All of the samples were run in triplicate to eliminate experimental error.

### Statistical analysis

At least three independent experiments were performed. The data are shown as the mean±s.e. and were analyzed for statistically significant differences using SPSS 19.0 software (SPSS Inc., Chicago, IL, USA). A Student’s *t*-test was used for comparisons between two groups. *P*<0.05 was considered statistically significant.

## RESULTS

### H1N1 infection of A549 and THP-1 cells induced nuclear translocation of HIF-1α but did not alter HIF-1α mRNA and total protein levels

**Figure 1:**
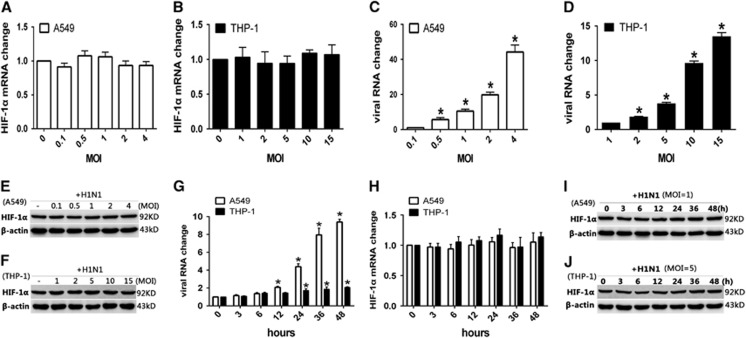
The change in HIF-1α mRNA and total protein levels in H1N1-infected A549 and THP-1 cells. (**A**–**F**) PBS (control) or A549 and THP-1 cells infected with H1N1 at MOIs of 0, 0.1, 0.5, 1, 2 and 4, and 0, 1, 2, 5, 10 and 15, respectively. After 24 h, the cells were collected. (**G**–**J**) A549 and THP-1 cells were treated with PBS (control) or infected with H1N1 at MOIs of 1 and 5, respectively. At 3, 6, 12, 24, 36 and 48 h, cells were collected. (**A**–**D**, **G** and **H**) Total cellular RNA was extracted and HIF-1α mRNA levels and viral RNA levels were measured by qRT-PCR. β-actin expression was used as an internal control. (**E**, **F**, **I** and **J**) Cell lysates were subjected to Western blot analysis using antibodies against HIF-1α. β-actin expression was used as a loading control. Data shown are the mean±SEM of three independent experiments. *P<0.05 compared to the first infected group.

To investigate the influence of H1N1 infection on the expression of HIF-1α, A549 and THP-1 cells were infected with H1N1 at different MOI values. Real-time quantitative RT-PCR and Western blot analysis were used to quantify the intracellular HIF-1α mRNA and protein levels, respectively. The results showed that HIF-1α mRNA expression was not upregulated in either A549 or THP-1 cells infected with H1N1 compared with the non-infected controls (Figures 1A and 1B), although the virus RNA levels increased with the infection dosage (Figures 1C and 1D). Similarly, the total protein level of HIF-1α was also not significantly changed in A549 (Figure 1E) or THP-1 cells (Figure 1F) at any infection dosage. We further measured the mRNA and protein levels of HIF-1α at different time points post-H1N1 infection. A549 and THP-1 cells were infected with H1N1 at MOIs of 1 and 5, respectively, and total RNA and protein samples were prepared at 3, 6, 12, 24, 36 and 48 h post infection. The intracellular viral RNA load increased gradually in A549 cells post-H1N1 infection, while in THP-1 cells, the viral RNA load was maintained at a relatively lower and more stable level throughout the observation time (Figure 1G). Nevertheless, the expression levels of intracellular HIF-1α mRNA (Figure 1H) and protein (Figures 1I and 1J) were constant.

**Figure 2:**
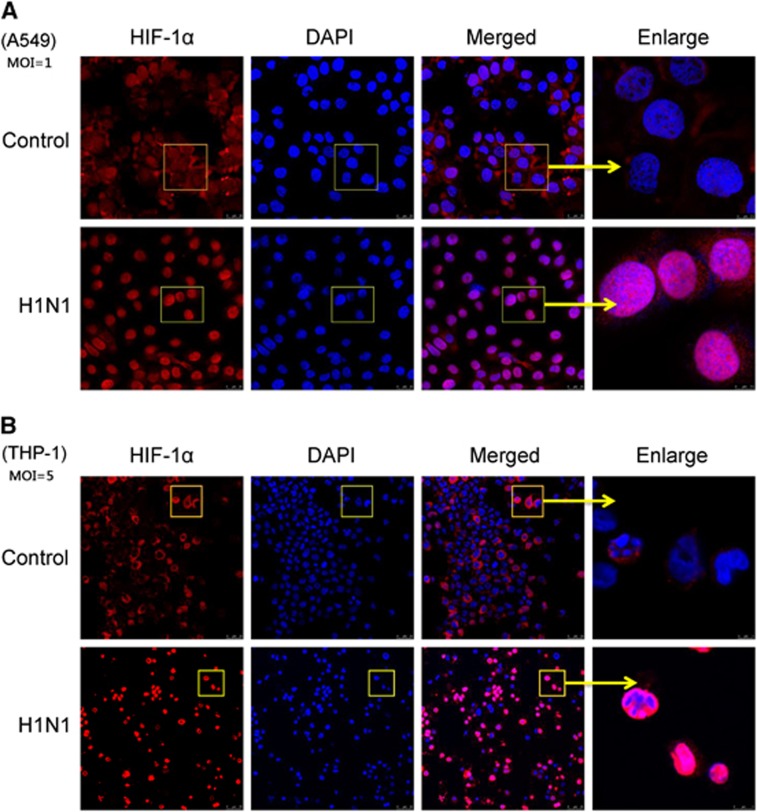
The nuclear accumulation of HIF-1α in H1N1-induced A549 and THP-1 cells. (**A** and **B**) A549 and THP-1 cells were treated for 24 h with PBS (control) or infected with H1N1 at MOI values of 1 and 5, respectively. Cells were incubated with anti-HIF-1α overnight at 4 °C and then stained with Alexa Fluor 555-Labeled Donkey Anti-Rabbit IgG for 1 h at room temperature. Nuclei were stained with DAPI. Immunofluorescence was observed and photographed by confocal microscopy. Magnification bar=25 μm. Yellow arrows indicate the enlargement of some cells, magnification bar=5 μm (THP-1 cells) and 7.5 μm (A549 cells). The results represent the findings from three independent experiments.

**Figure 3:**
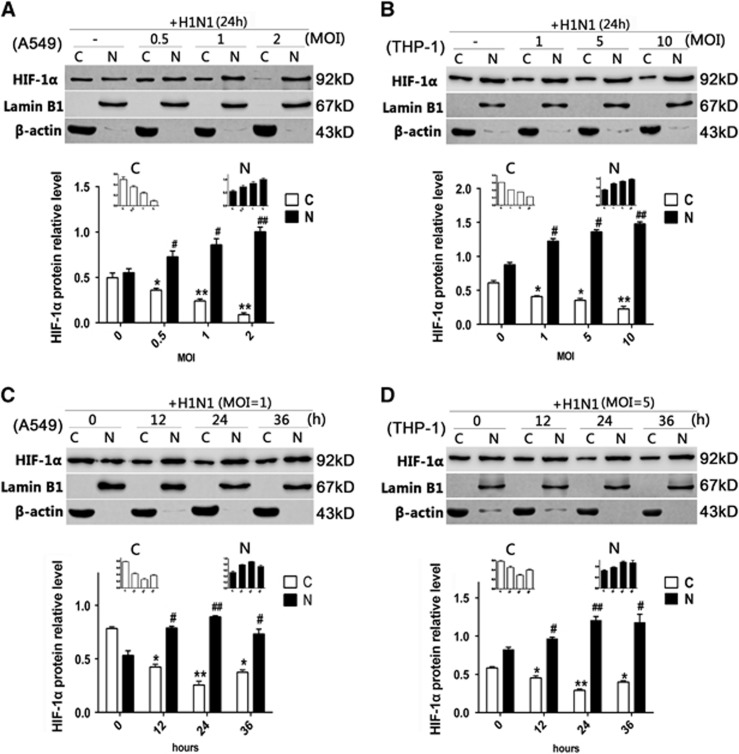
The cytoplasmic and nuclear protein levels of HIF-1α in H1N1-infected A549 and THP-1 cells. (**A** and **B**) A549 and THP-1 cells were treated with PBS (control) or infected with H1N1 at MOI values of 0, 0.5, 1 and 2, and 0, 1, 5 and 10, respectively. After 24 h, cells were collected. (**C** and **D**) A549 and THP-1 cells were treated with PBS (control) or infected with H1N1 at MOIs of 1 and 5, respectively. After 12, 24 or 36 h, cells were collected. Nuclear and cytoplasmic extraction reagents were used to extract the nuclear and cytoplasmic proteins from the cells. Western blot analysis was performed. β-actin was used as a loading control of cytoplasmic protein (abbreviated as ‘C’ in the figure), and lamin B1 was used as a loading control of nuclear protein (abbreviated as ‘N’ in the figure). The intensities of the protein bands from three typical experiments were quantified using Image J software. The data are shown as the mean±SEM of three independent experiments. *P<0.05, **P<0.01 compared with the cytoplasmic protein from the control group. ^#^*P*<0.05, ^##^*P*<0.01 compared with the nuclear protein from the control group.

Since HIF-1α belongs to the bHLH-PAS protein family and the accumulation of HIF-1α in the nucleus might regulate its target genes,^15^ an immunofluorescence assay was used to confirm whether HIF-1α protein accumulated in the nucleus post-H1N1 infection. Without H1N1 infection, HIF-1α was scarcely visible in the nucleus of A549 cells and was mainly observed in the cytoplasm. However, after H1N1 infection, a strong nuclear signal of HIF-1α was detected (Figure 2A). A similar phenomenon was also observed in THP-1 cells (Figure 2B). To further confirm these observations, the nuclear and cytoplasmic HIF-1α protein levels were analyzed by Western blot. In both A549 and THP-1 cells, the level of cytoplasmic HIF-1α protein decreased, while the level of nuclear HIF-1α protein increased in a dose-dependent manner (Figures 3A and 3B). To illustrate the kinetics of the nuclear accumulation of HIF-1α, cells were collected at different time points post virus infection. Both in A549 and THP-1 cells, the cytoplasmic HIF-1α level declined and the nuclear HIF-1α level increased from 12 to 36 h post infection (*P*<0.05; Figures 3C and 3D). The peak relative level of nuclear HIF-1α was observed 24 h post infection (*P*<0.01, Figures 3C and 3D). These data indicated that H1N1 infection induced the nuclear translocation of HIF-1α.

### Blocking the nuclear accumulation of HIF-1α reduced pro-inflammation cytokine secretion in both H1N1-infected A549 and THP-1 cells

**Figure 4:**
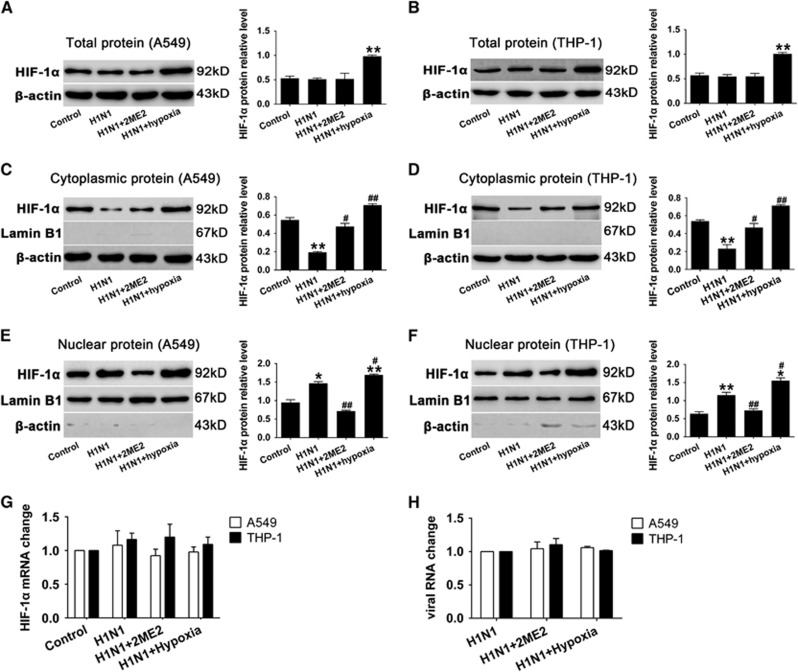
Effect of 2ME2 and hypoxia on HIF-1α nuclear accumulation in H1N1 infection. Before cells were infected with H1N1 at multiplicity of infection (MOIs) of 1 and 5 for 24 h, A549 and THP-1 cells were pretreated with 0.1 and 0.2 μM 2ME2 for 30 min. A549 and THP-1 cells were exposed to 1% O_2_ for 24 h after H1N1 infection. (**A** and **B**) Cells were lysed using RIPA buffer to extract the total protein. (**C**–**F**) Nuclear and cytoplasmic extraction reagents were used to extract the nuclear and cytoplasmic proteins from the cells. Western blots were performed. β-actin was used as a loading control for cytoplasmic protein and lamin B1 was used as a loading control for nuclear protein. The intensity of the protein bands from three typical experiments was quantified using Image J software. (**G**, **H**) Total cellular RNA was extracted and HIF-1α mRNA levels and viral RNA levels were measured by qRT-PCR. β-actin expression was used as an internal control. Data are shown as the mean±s.e.m. of three independent experiments. *P<0.05, **P<0.01 compared with the control group. ^#^*P*<0.05, ^##^*P*<0.01 compared with the H1N1-infected group.

To investigate the role of HIF-1α nuclear translocation during H1N1 infection, 2ME2 was used to inhibit HIF-1α nuclear accumulation.^27^ A549 and THP-1 cells were pretreated with 2ME2 for 30 min before H1N1 infection. Our data showed that 2ME2 did not significantly alter the total protein levels of HIF-1α in A549 and THP-1 cells (*P*>0.05, Figures 4A and 4B). However, it increased the cytoplasmic level of HIF-1α compared to PBS-treated H1N1-infected cells (*P*<0.05; Figures 4C and 4D), and blocked HIF-1α nuclear accumulation (*P*>0.05; Figures 4E and 4F). In addition, the level of HIF-1α mRNA (Figure 4G) and the replication of H1N1 (Figure 4H) did not significantly change among groups (*P*>0.05). The results showed that 2ME2 blocked the translocation of HIF-1α in H1N1-infected cells.

**Figure 5:**
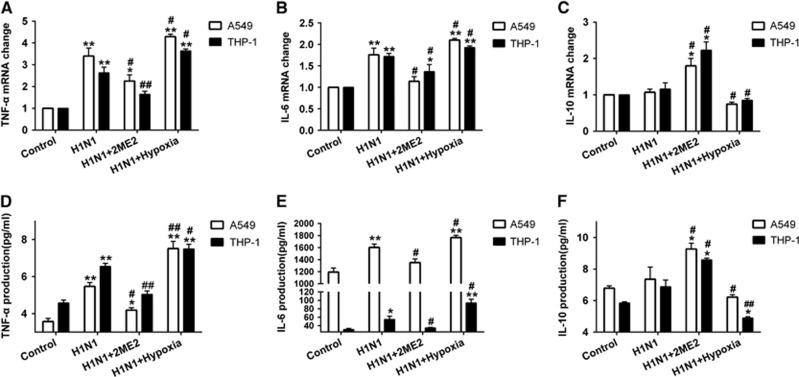
Effect of 2ME2 and hypoxic conditions on the expression of TNF-α, IL-6, and IL-10 in H1N1-infected cells. Before A549 and THP-1 cells were infected for 24 h with H1N1 at MOIs of 1 and 5, respectively, they were pretreated with 0.1 and 0.2 μM 2ME2 for 30 min. A549 and THP-1 cells were exposed to 1% O_2_ for 24 h after H1N1 infection. qRT-PCR was performed to measure mRNA levels of TNF-α (**A**), IL-6 (**B**) and IL-10 (**C**). ELISA was performed to detect the levels of TNF-α (**D**), IL-6 (**E**) and IL-10 (**F**) in cell supernatants. The data are shown as the mean±s.e.m. of three independent experiments. *P<0.05, **P<0.01 compared with the control group. ^#^*P*<0.05, ^##^*P*<0.01 compared with the H1N1-infected group

To investigate the role of HIF-1α nuclear accumulation in H1N1 infection-induced inflammation and cytokine secretion, cytokine secretion was determined during H1N1 infection with or without 2ME2. H1N1 infection increased TNF-α and IL-6 mRNA levels more than onefold when compared with control cells (*P*<0.01; Figures 5A and 5B). After pre-treatment with 2ME2, TNF-α and IL-6 mRNA levels were lower than those in the H1N1-infected group (*P*<0.05; Figures 5A and 5B). The level of IL-10 mRNA was not enhanced in the H1N1-infected group (*P*>0.05; Figure 5C). When compared to the H1N1-infected group, the 2ME2 group had more than a onefold (*P*<0.05) increase in IL-10 mRNA expression. Moreover, TNF-α (Figure 5D), IL-6 (Figure 5E) and IL-10 (Figure 5F) protein expression in cell supernatants was similar in pattern to the mRNA expression. These data suggest that blocking HIF-1α nuclear accumulation with 2ME2 reduced the levels of the proinflammatory cytokines TNF-α and IL-6 and elevated the levels of the anti-inflammatory cytokine, IL-10.

### Increasing HIF-1α nuclear accumulation by hypoxia condition led to enhanced proinflammatory cytokine secretion both in H1N1-infected A549 and THP-1 cells

To investigate the effects of increasing HIF-1α nuclear accumulation, cells were infected with H1N1 and then incubated in a hypoxic condition of 1% O_2_ for 24 h. The data showed that the expression of HIF-1α was significantly increased (*P*<0.01; Figures 4A and 4B) under this hypoxic condition. A combination of H1N1 infection and hypoxia significantly increased HIF-1α expression in both the cytoplasm (*P*<0.01; Figures 4C and 4D) and nucleus (*P*<0.05, Figures 4E and 4F), which indicated that, in the context of H1N1 infection, hypoxia exacerbated HIF-1α nuclear accumulation through the induction of HIF-1α protein expression. Furthermore, we detected the levels of inflammatory cytokine secretion in H1N1-infected, hypoxic cells. As a result, H1N1 infection with hypoxia conditions enhanced the expression of TNF-α and IL-6 (*P*<0.05, Figures 5A, 5B, 5D and 5E) and decreased the expression of IL-10 (*P*<0.05, Figures 5C and 5F) in comparison with the H1N1-infected group. These data suggest that hypoxia enhances HIF-1α nuclear accumulation and leads to increased TNF-α and IL-6 secretion and reduced IL-10 levels.

## DISCUSSION

HIF-1 is a heterodimeric transcription factor, and its regulatory function is mainly controlled by HIF-1α, which is the oxygen sensitive subunit. Many studies have shown that HIF-1α activity is affected by virus infection.^23, 24, 25, 26, 28^ However, whether HIF-1α takes part in influenza A infection is still unknown. As far as we know, only one study has focused on HIF-1α in influenza A virus infection. The study reported that HIF-1α was upregulated in the lungs of H5N1-infected cynomolgus macaques, which suggested that measurement of HIF-1α expression could be used as a prognostic biomarker in severe respiratory infections.^29^ Considering hypoxia occurs in lung tissue with severe inflammation caused by influenza virus infection,^30, 31^ a potential explanation for the increased levels of HIF-1α observed *in vivo* is that they may be induced by hypoxia.^32, 33, 34^ In our study of A549 and THP-1 cells cultured under normal oxygen concentrations, H1N1 infection did not stimulate the expression of HIF-1α, but it did promote the nuclear translocation of HIF-1α. However, if virus infection was combined with hypoxia, the expression of HIF-1α was upregulated and nuclear translocation of HIF-1α was enhanced, thereby inducing higher levels of proinflammatory cytokines. The data here suggest that viral infection and hypoxia play important roles in the development of severe inflammation during H1N1 infection.

HIF-1α plays an important role in regulating the expression levels of cytokines such as TNF-α, IL-10 and IL-6.^35, 36^ After HIF-1α nuclear accumulation was inhibited, the expression of TNF-α and IL-6 was positively associated with the nuclear protein levels of HIF-1α in H1N1 infection, while the expression of IL-10 was negatively correlated. These data show that the nuclear accumulation of HIF-1α may be involved in the regulation of cytokine expression in H1N1 infection, which is concordant with data from other studies.^37, 38, 39, 40^ It could be concluded that HIF-1α translocation played an important role in development of inflammation in H1N1 infection.

However, the mechanism of HIF-1α translocation induced by H1N1 infection is still unknown and is a complex process. Previous research has shown that HIF-1α is regulated by O_2_-dependent mechanisms and O_2_-independent mechanisms.^41^ Under hypoxic conditions, the activity of prolyl hydroxylases is inhibited, which can target HIF-1α for proteasomal degradation, resulting in increased levels of HIF-1α protein and enhanced transcription of HIF-1α target genes. When the O_2_ level is normal, bacterial or viral infections can upregulate HIF-1α mRNA and protein levels.^23, 24, 25, 26, 42^ Interestingly, we found that H1N1 infection did not alter HIF-1α mRNA or total protein expression, but it did induce nuclear translocation of HIF-1α under normal oxygen concentrations. Previous studies have shown that importin α/β mediates the nuclear–cytoplasmic transportation either for cellular proteins or viral proteins.^43, 44^ Importins 4 and 7 are required for nuclear translocation of HIF-1α.^45^ On the other hand, influenza virus polymerase recruits importin-α1 and -α7.^46^ Thus, we speculated that H1N1 infection might directly recruit the factors that transport HIF-1α into the nucleus resulting in cytokine expression. Further studies are required to support this result.

**Figure 6:**
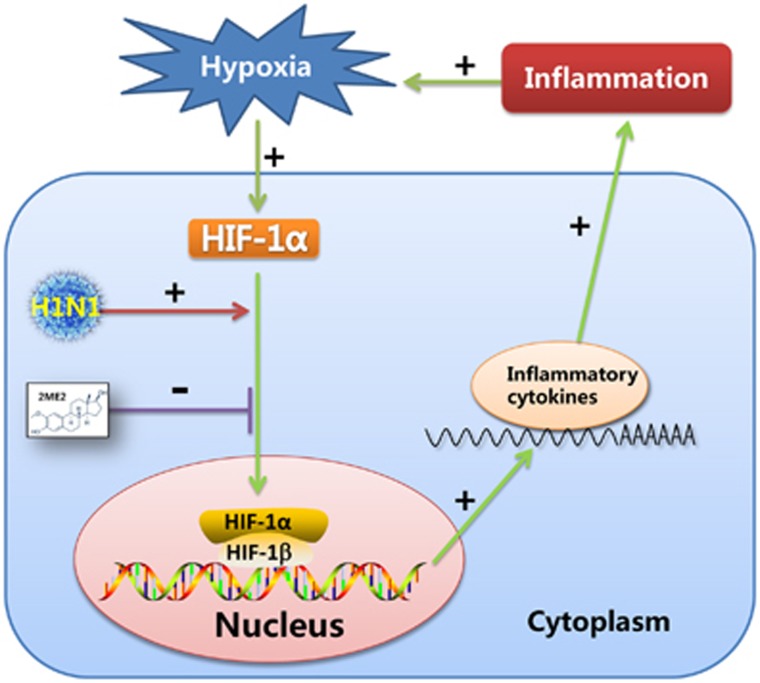
Schematic model of the possible mechanism of HIF-1α involvement in H1N1 infection-induced inflammation. In this process, the nuclear translocation of HIF-1α, induced by H1N1 infection, might promote the secretion of proinflammatory cytokines in H1N1 infection. Alveolar hypoxia induced by lung inflammation, upregulates HIF-1α protein expression. Importantly, the nuclear translocation of HIF-1α induced by H1N1 infection plays an important role in this vicious cycle.

Recent studies have demonstrated that proinflammatory effect of pulmonary hypoxia is important.^47, 48, 49, 50^ It is generally accepted that hypoxia can promote inflammation through the HIF pathway.^51, 52^ On the other hand, microenvironmental hypoxia can be induced by the development of inflammation, which increases inflammatory cell infiltration and exudation of protein-rich edema fluid and fibrin.^32^ The results of our research supported the following scenario. H1N1 infection induced the nuclear translocation of HIF-1α without altering HIF-1α mRNA or total protein expression under normal oxygen concentrations, and HIF-1α nuclear accumulation enhanced the secretion of proinflammatory cytokines which, *in vivo*, could result in inflammation in the lung tissue. With inflammatory diffusion, microenvironmental hypoxia could be induced, which would strongly increase the level of HIF-1α and be one of the drivers of HIF-1α nuclear accumulation. Thus, an irreversible, vicious cycle would be created between H1N1 infection and inflammation, which may contribute to H1N1-induced, severe pneumonia. Above all, the nuclear translocation of HIF-1α induced by H1N1 infection may play an important role in this cycle (Figure 6).

Some drugs attenuate inflammation by suppressing the expression or activity of HIF-1α, which suggests that HIF-1α is an important new therapeutic target in control of inflammation.^53, 54, 55^ In our study, suppressing H1N1-induced HIF-1α nuclear accumulation using 2ME2, which blocks HIF-1α nuclear accumulation by depolymerizing microtubules,^27^ decreased proinflammatory cytokine secretion, which may attenuate inflammation in H1N1 infection (Figure 6). There is still a lack of effective drugs to control influenza-induced, severe pneumonia. Early treatment with antiviral drugs alleviates the symptoms, but the effect of treatment with antiviral drugs in influenza-induced severe pneumonia is limited and there is increasing drug resistance in antiviral therapy.^56^ On the other hand, immunomodulatory therapies, such as the use of corticosteroids, do not prevent the development of severe pneumonia and is associated with a risk of superinfections.^57^ Therefore, finding new drug targets has become an urgent problem that needs to be addressed. Recent studies reported some drug targets were possibly effective to control cytokine storm. For example, sphingosine-1-phosphate receptor (S1PR) agonist therapy can ameliorate the cytokine storm.^58^ Mint3/Apba3 may be a therapeutic target for the treatment of severe influenza pneumonia, because Mint3/Apba3 decreased cytokine/chemokine production in response to influenza virus infection by inactiving HIF-1.^59^ In our study, HIF-1α may be a potential target for the development of drugs to treat severe pneumonia caused by the influenza virus.

In conclusion, *in vitro* H1N1 infection under normal oxygen concentrations induced nuclear translocation of HIF-1α instead of altering its expression. It might promote the proinflammatory cytokine secretion in H1N1 infection, which suggests that HIF-1α might be a target for the treatment of H1N1 infection-induced, severe pneumonia. However, there are still limitations to this research because it is an *in vitro* assay and further *in vivo* research is needed to fully understand the mechanism by which H1N1 infection induces HIF-1α nuclear translocation.
