# Supplementary material for: Nuclear translocation of HIF-1α induced by influenza A (H1N1) infection is critical to the production of proinflammatory cytokines
Source: Emerg Microbes Infect. 2017 May 24;6(5):e39–. doi: 10.1038/emi.2017.21 (PMC5520484; doi:10.1038/emi.2017.21)
Supplement: Supplementary Table S1 [file emi201721x1.docx]

| Target | Probe/Primer | Sequence |
| --- | --- | --- |
| HIF-1α | Probe | 5'-(FAM)-CCTCTTCGTCGCTTCGGCCAGTGT-(TAMRA)-3' |
|  | F | GTCTGAGGGGACAGGAGGAT |
|  | R | CTCCTCAGGTGGCTTGTCAG |
|  |  |  |
| M | Probe | 5'-(FAM)-TGCAGTCCTCACTGGGCACG-(TAMRA)-3' |
|  | F | 5'-GACCRATCCTGTCACCTCTGAC-3' |
|  | R | 5'-AGGGCATTYTGGACAAAKCGTCTA-3' |
|  |  |  |
| TNF-α | Probe | 5'-(FAM)-ATCAGCCGCATCGCCGTCTCCTACC-(TAMRA)-3' |
|  | F | 5'-GCCTGTACCTCATCTACTCC-3' |
|  | R | 5'-CAGATAGATGGGCTCATACCAG-3' |
|  |  |  |
| IL-6 | Probe | 5'-(FAM)-CTCTGGCTTGTTCCTCACTACTCTCAA-(TAMRA)-3' |
|  | F | 5'-GAGGAGACTTGCCTGGTGAA-3' |
|  | R | 5'-CTGCAGGAACTGGATCAGGACT-3' |
|  |  |  |
| IL-10 | Probe | 5'-(FAM)-TTGCCAAGCCTTGTCTGAGATGATCCAGTT-(TAMRA)-3' |
|  | F | 5'-CTGGAGGACTTTAAGGGTTAC-3' |
|  | R | 5'-TTGATGTCTGGGTCTTGGTT-3' |
|  |  |  |
| β-actin | Probe | 5'-(FAM)-ACAACAATGTGCAATCAAAGTCCTCGGC-(TAMRA)-3' |
|  | F | 5'-CTGGAACGGTGAAGGTGACA-3' |
|  | R | 5'-AAGGGACTTCCTGTAACAATGCA-3' |

**Supplementary Table S1 Quantification was performed using a Real Time One Step RT-PCR protocol as described in the manual**. The protocol consisted of 1 cycle at 42°C for 5 min and then 95°C for 10 s, 40 cycles at 95°C for 5 s, and a final step at 60°C for 31 s; fluorescence was monitored at this step.
